# Ethnobotanical Insights into Medicinal and Culinary Plant Use: The Dwindling Traditional Heritage of the Dard Ethnic Group in the Gurez Region of the Kashmir Valley, India

**DOI:** 10.3390/plants12203599

**Published:** 2023-10-17

**Authors:** Laraib Ahad, Musheerul Hassan, Muhammad Shoaib Amjad, Rayees Afzal Mir, Ivana Vitasović-Kosić, Rainer W. Bussmann, Zakia Binish

**Affiliations:** 1 School of Agricultural Sciences, Glocal University, Saharanpur 247121, Uttar Pradesh, India; laraibahad@gmail.com (L.A.); ubaidshafi_8@hotmail.com (R.A.M.); 2 Department of Ethnobotany, Institute of Botany, Ilia State University, Tbilisi 0162, Georgia; musheer123ni@gmail.com (M.H.); rainer.bussmann@smnk.de (R.W.B.); 3 Alpine Institute of Management and Technology, Nanda Ki Chowki, Dehradun 248007, Uttarakhand, India; 4 Department of Botany, Women University of Azad Jammu & Kashmir Bagh, Bagh 12500, Pakistan; zakiabenish88@gmail.com; 5 Birmingham Institute of Forest Research, University of Birmingham, Birmingham B15 2TT, UK; 6 Division of Horticulture and Landscape Architecture, Department of Agricultural Botany, Faculty of Agriculture, University of Zagreb, Svetošimunska cesta 25, 10000 Zagreb, Croatia; ivitasovic@agr.hr; 7 Department of Botany, State Museum of Natural History, 76133 Karlsruhe, Germany

**Keywords:** Dard, ethnopharmacology herbal tradition, ethno-food, traditional medicinal praxis

## Abstract

This ethnobiological study addresses the complicated relationship between the Dard ethnic group and their natural environment in the Gurez region of the Kashmir Valley. The study documents their traditional knowledge of the use of plant species for medicinal and culinary purposes. A total of 87 plant species from 41 different families were cataloged, with the Asteraceae family (15 species) and the Lamiaceae family (12 species) being the most commonly used. These plants were found to be used to treat 20 different ailments, with menstrual cramps being the most common (12 species). The fidelity values for these plants ranged from 11.10 to 71.42, demonstrating their importance in traditional medicine. In addition, 17 plant species were found to be useful for gastronomic purposes, with *Juglans regia* being the most valuable (use value of 0.73). The study also evaluated the conservation status of these plants and found that seven of them are considered critically endangered, ten endangered, and four endangered according to the IUCN classification. This study offers insights into the Dard people’s deep connection to their natural environment and has significant implications for policy formulation, cultural conservation, and sustainable use of endemic species, as well as potential applications in pharmaceutical research for therapeutic compounds.

## 1. Introduction

Humans have known of the therapeutic properties of plants since the beginning of their evolutionary history, as reflected in their prehistoric and later cultural heritage [1,2,3]. In all ethnic communities, conventional medicine is considered the sum of knowledge. Human talents and cultural practices of a society based on its beliefs, experiences, and theories are used to treat or improve health [4]. Modern medicines produced by chemical synthesis are far more accessible in developed countries, although in many cases they are based on a molecule of natural origin (plant or animal) [5]. Nevertheless, developed countries increasingly value the direct use of herbs in conjunction with modern medical treatments, especially herbs with a scientific basis for treating minor illnesses [6]. Developing countries continue to rely on the use of medicinal herbs, and this is despite the fact that traditional knowledge is being lost in many societies [7]. Traditional medicine systems are extremely effective in treating various common seasonal diseases [8]. However, this traditional knowledge gathered in traditional medicine and ethnopharmacology is declining and has been severely threatened in recent decades. Numerous ethnobiologists believe that this valuable knowledge could be extinguished by the end of time [9]. Plants as traditional remedies are a real option for health care in developing countries, especially for rural communities [10].

About 2.5 million years ago, the human lineage transitioned from a predominantly vegetarian lifestyle [11]. The Himalayan region, known for its ecological richness, hosts more than half of India’s biodiversity and is characterized by a large number of rare and endemic species that are an important source of food [12]. Ethnobiology has evolved considerably in recent decades, moving from mere documentation to practical application and sustainable management of traditional knowledge systems. This evolution is in line with Article 8 (j) of the Convention on Biological Diversity (CBD), which explicitly recognizes traditional knowledge (TK) as a cornerstone for the sustainable development of food systems in a given geographic context [13]. Traditional plant gathering is critical to the creation of new local gastronomies and the sustainability of food systems in remote tribal communities [14,15]. Food scouting, the identification, classification, and exchange of a range of food resources within indigenous communities, is enabled by ethnobiological studies [15,16]. According to such studies, indigenous peoples have a great treasure of lost plant and ecological knowledge that needs to be documented in a timely manner to build sustainable food and healthcare systems [17,18,19].

Ethnopharmacology is defined as the interdisciplinary scientific study of traditionally used indigenous drugs and biologically active agents [20,21,22]. A first step is to present the use of extracts in a particular disease without investigating a possible causal relationship with the contained ingredients/active ingredients [22]. To date, ethnopharmacology has contributed significantly to the study of indigenous and traditional medicinal knowledge and the biodiversity component with which this knowledge is associated [3]. Ethnomedicine is a traditional ethnic approach to treating health problems using plants or other natural sources [23,24]. In the Kashmir Valley, a spectrum of ethnic communities has been ethnobotanically studied, although the use of plants for medicinal and dietary purposes by the “Dard” ethnic group remains relatively unexplored. Several factors could explain the limited research efforts in this population group. These include their residence in remote, high-altitude areas with inadequate road infrastructure, cultural constraints that discourage women from participating in studies, and diminished confidence due to regional geopolitical challenges. Against this background, this study was designed to systematically document ethnomedical knowledge in the Gurez (Kashmir Valley) of India. The objectives of the study were as follows: (A) to document the plant species used to control various ailments and (B) to record the plant species consumed as preferred foods at different stages of pregnancy. This research will help collect baseline data from the Gurez region of the Kashmir Valley for pharmaceutical companies to identify new compounds with significant therapeutic properties.

## 2. Results and Discussion

### 2.1. Ethnobotanical Inventory

**Figure 1:**
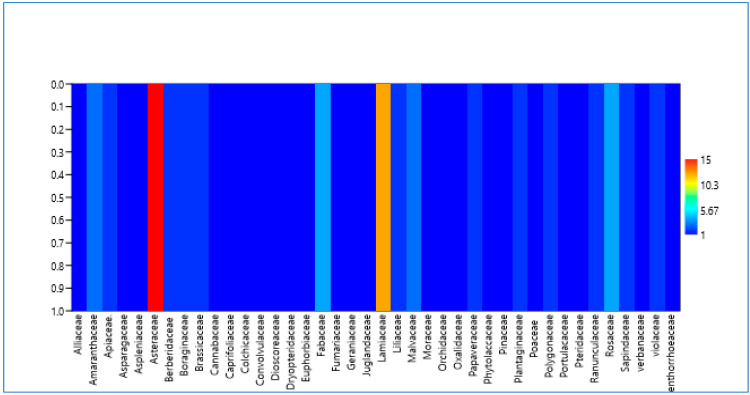
Matrix plot showing the species family relationship of the documented species in Kashmir Himalaya.

**Figure 2:**
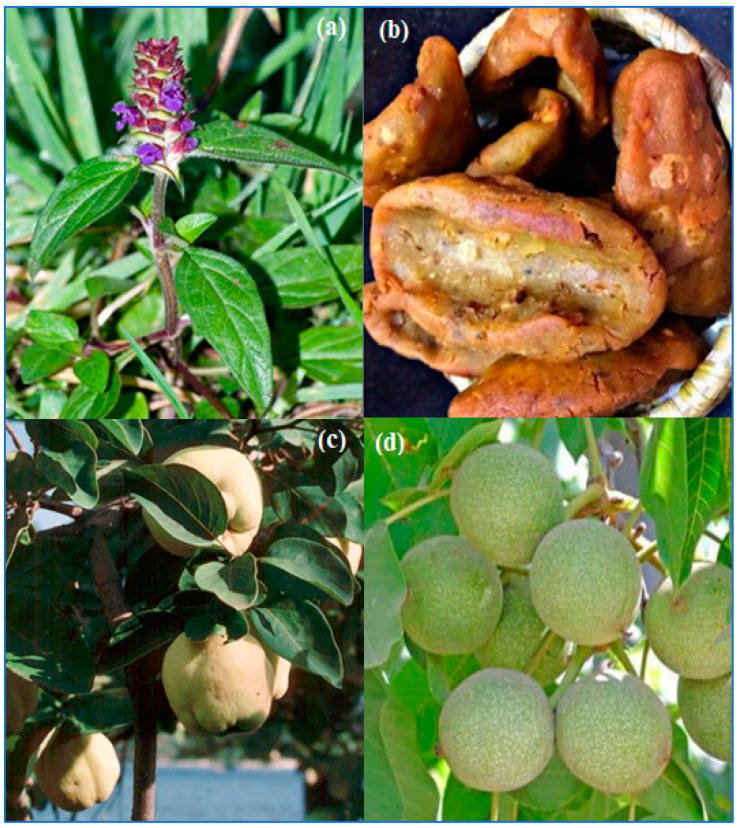
Some pictures taken during the study period in Kashmir Himalaya (**a**) *Prunella vulgaris*; (**b**) Yaja (made from walnut flour); (**c**) *Cydonia oblonga*; (**d**) *Juglans regia*.

In the present study, a total of *n* = 87 species were recorded, divided into *n* = 41 families. Asteraceae was the dominant family with *n* = 15 species, followed by Lamiaceae (*n* = 12), Fabaceae, Rosaceae (*n* = 4 each), Amaranthaceae, and Malvaceae (*n* = 3 each) (Figure 1). The dominance of Asteraceae can be attributed to their rapid acclimation and adaptation to dry sites due to their large ecological amplitude [25]. Based on growth habit, species were classified into trees, herbs, and shrubs, with herbs (*n* = 76) taking potential precedence, followed by trees (*n* = 7) and shrubs (*n* = 4) (Table 1). The predominance of herbs can be attributed to their frequent distribution, ease of collection, and rich phytochemistry [26]. The present study is the first comprehensive investigation of ethnobotanical findings on the use of plant species for medicinal and dietary purposes within the Dard ethnic group. The data obtained in the study are shown in Table 1, and some pictures of the species are shown in Figure 2.

### 2.2. Ethnopharmacological Profile

**Figure 3:**
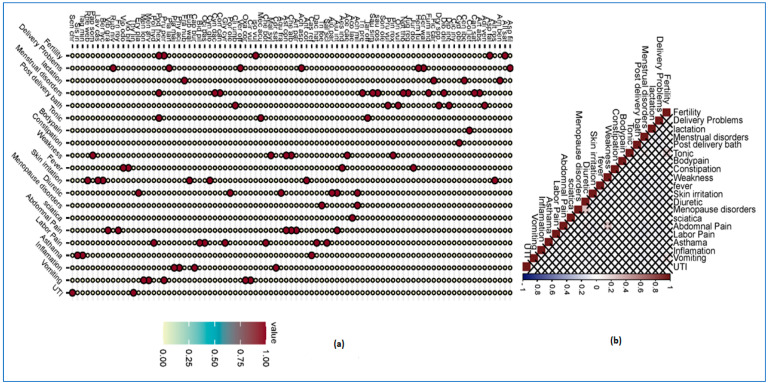
(**a**) Balloon plot showing species used against different diseases; (**b**) Cladogram showing Pearson correlation between plant species and ailments. The full names of the species are given in Table 1.

The use of plant taxa for a variety of ailments is common in different cultures around the world. In the present study, we documented *n* = 20 diseases treated with the documented species (Figure 3a). Most (*n* = 12) of the species (*Artemisia absinthium*, *Cannabis sativa*, *Dioscorea deltoidea*, *Fumaria indica*, *Juglans regia*, *Notholirion thomsonianum Aucklandia costus*, *Saussurea simpsoniana*, *Trifolium pratense*, *Erigeron canadensis*, *Cynodon dactylon*, *Podophyllum hexandrum*) were used for menstrual disorders, followed by delivery problems (*n* = 7; i.e., *Asparagus filicinusa*, *Geranium wallichianum*, *Hemerocallis fulva*, *Achyranthes aspera*) and labor pain (*n* = 7; *Salvia sclarea*, *Dactylorhiza hatagirea*, *Dyspania botrys*, *Glycyrrhiza glabra*, *Ocimum basilicum*, *Bidens pilosa*, *Persicaria hydropiper*) (Figure 3a). The use of the above species against the selected diseases can be attributed to the strong belief in traditional knowledge. The local Dards believe that sources from nature have healing properties because they are blessed with divine stature that mankind cannot even think of. The results are in line with many other studies [23,27,28] from the nearby Himalayan region, which show that plant taxa still play an important role (health care) even in modern times. It is important to mention that poor medical facilities in the region and being cut off from the other parts of the state due to heavy snowfall make people invest their minds in nature to maintain their health. Table 1 provides a detailed overview of the use of the documented taxa against a variety of diseases. The Pearson correlation coefficient highlights the strength and direction of the relationship between the diseases and the documented species; *p-*values are shown below (Figure 3b).

**Table 1:** Taxonomic inventory and ethnomedicinal use of documented species of the ethnic community (Dard) in Kashmir Himalaya.

Scientific Name (Family) Voucher Number	Abre	Common Name (Local Name)	Habit	Part Used	Ethno-Medicinal Uses					IUCN Status
					Preparation	Ailments	Np	F_C_	FL	
*Asparagus filicinus* Buch.-Ham. ex D.Don (Asparagaceae) LAB.203	Asp fil	Fern (Parglas)	Herb	Seed	Seeds are boiled in water to produce decoction, given in the last month of pregnancy.	Body pain	3	11	27.27	TH
*Astragalus confertus* Benth.ex Bunge. (Fabaceae) LAB.515	Ast con	Hairy-leaved milk vetch (Vetch)	Herb	Roots	Roots are dried and powdered and consumed with mint and water.	Abdominal pain	3	8	37.50	DD
*Aquilegia fragrans* Benth. (Ranunculaceae) LAB.516	Aqu fra	Fragrant columbine (Jangli kuth)	Herb	Roots	Roots are crushed to powder and given orally.	Diuretic	2	6	33.33	CR
*Allium sativum* L. (Amaryllidaceae) LAB.204	All sat	Garlic (Rhoon)	Herb	Stem	Stem is consumed raw.	Menstrual issue	15	24	62.5	LC
						Fertility problems	8		33.32	
*Arnebia benthamii* (Wall.ex G.Don) I.M.Johnst (Boraginaceae) LAB.205	Arn ben	Himalayan Arnebia (Kahzabaan)	Herb	Whole plant	The roots are crushed and boiled in water. The decoction is taken orally.	Lactation problem (Hypogalactia)	4	11	36.36	CR
*Alcea rosea* L. (Malvaceae) LAB.206	Alt ros	Hollyhocks (Sazeposh)	Herb	Flower	The flowers are boiled to prepare decoction taken orally.	Skin irritation	3	14	21.42	LC
*Asplenium falcatum* Lam. (Aspleniaceae) LAB.207	Asp fal	Holly fern (Dade)	Herb	Whole plant	The plant is cooked and used as a vegetable.	Fertility problems	2	13	15.38	VU
*Adiantum venustum* D.Don. (Pteridaceae) LAB.208	Adi ven	(Himalayan maiden fern) (Kakbai)	Herb	Whole plant	Whole plant is crushed and boiled in water.	Used for bath after delivery	1	7	14.28	EN
*Artemisia absinthium* L. (Asteraceae) LAB.209	Art abs	Wormwood (Tethwan)	Herb	Leaves	Dried leaves after soaking in hot water are dried and then made into tablets, taken orally.	Prevent excessive menstrual bleeding	6	14	42.85	EN
*Achillea millefolium* L. (Asteraceae) LAB.501	Ach mil	Devil’s nettle (Pahel ghass)	Herb	Whole plant	Leaves are made into decoction and consumed empty stomach.	Menopause disorders	3	14	21.42	LC
*Aconitum laeve* Royle. *(Ranunculaceae)* LAB.502	Aco.lae	Grape-leaved monkshood (Patrees)	Herb	Roots	Roots are dried, powdered, and consumed with milk.	Sciatica	3	11	27.27	EN
*Acer caesium* Wall. ex. Brandis. *(Sapindaceae)* LAB.503	Ace.cae	Himalayan Maple (Chhad)	Tree	Leaves	Leaves are boiled in water and taken orally.	Weakness	2	6	33.32	LC
*Aesculus indica* (Wall.ex Cambess) Hook (Sapindaceae) LAB.504	Aes ind	Indian Horse-Chest nut (Handoon)	Tree	Leaves	Extract from leaves is consumed with lukewarm water.	Fever	2	8	25	LC
*Ajuga intregrifolia* Buch.-Ham. (Lamiaceae) LAB.505	Aju int	Bracted Bugleweed (Jani Adam)	Herb	Whole plant	Decoction is obtained from fresh plant and consumed empty stomach to remove toxins from the body by acting as a diuretic.	Diuretic	7	13	53.84	-----
*Ajuga parviflora* Benth. (Lamiaceae) LAB.506	Aju par	Small-Flowered Bugleweed (Jangli Jani-e- Adam)	Herb	Leaves	Extract of leaves is mixed with a glass of water and sugar, taken orally early morning, which in turn causes frequent urination.	Diuretic	2	6	33.32	VU
*Anthemis cotula* L. (Asteraceae) LAB.511	Ant cot	Stinking Chamomile (Fakghass)	Herb	Whole plant	Whole plant is made into a paste and applied topically.	Skin irritation	3	8	37.5	LC
*Achyranthes aspera* L. (Amaranthaceae) LAB.512	Ach asp	Chaff flower (Puthkanda)	Herb	Root	Roots are crushed to powder and given orally with water in the last month of pregnancy.	Delivery issue	5	13	38.46	LC
*Artemisia parviflora* Roxb.ex D.Don (Asteraceae) LAB.513	Art par	Himalayan Worm Wood (Tethwan)	Herb	Whole plant	Extract is obtained and given orally.	Abdominal pain	5	9	55.55	DD
*Aucklandia costus* Falc. (Asteraceae) LAB.229	Auc cos	Costus (Kouth)	Herb	Root	Roots are crushed into a powder and drunk with water.	Menstrual issue	3	7	42.85	CR
*Berberis lycium* Royle (Berberidaceae) LAB.550	Ber lyc	Indian Barberry (Kawdach)	Shrub	Whole plant	Ripened barriers are applied topically.	Skin irritation	3	16	18.75	TH
*Bidens pilosa* L. (Asteraceae) *LAB.532*	Bid pil	Begger’stick (Kumber)	Herb	Leaves	Leaves are made into decoction and given orally.	Labor pain	2	6	33.32	NE
*Cannabis sativa* L. (Cannabaceae) LAB.210	Can sat	Hemp, Gallow Grass (Bhang)	Herb	Leaves	The leaves are crushed and made into powder, mixed with cow ghee to make paste taken orally.	Menstrual pain	1	7	14.28	VU
*Colchicum luteum* Baker (Colchicaceae) LAB.211	Col let	Meadow Saffron (Veir koem)	Herb	Stem	The stem is crushed to form a paste and applied to feet.	Relieve body pain in fresh mothers	2	9	22.22	TH
*Centaurea iberica* Trevir.ex Spreng. (Asteraceae) LAB.212	Cen ibe	Iberian Star thistle (Kreaxeh)	Herb	Leaves	Fresh leaves after crushing are mixed with egg and then cooked to prepare an omelet.	Lactation problem (hypogalactia)	1	8	12.5	NT
*Cydonia oblonga* Mill. (Rosaceae) LAB.213	Cyd obl	Quince (Bum chount)	Tree	Seeds	Seeds are made into infusion, taken orally.	Constipation	4	9	44.43	LC
*Cichorium intybus* L. (Asteraceae) LAB.214	Cic iny	Chicory (Posh handh)	Herb	Leaves	Leaves are cooked.	Easy delivery	2	11	18.18	LC
*Cuscuta reflexa* Roxb. (Convolvulaceae) LAB.510	Cus ref	Gaint Dodder (Kukli-Poot)	Herb	Whole plant	Decoction is prepaid by boiling the whole plant, kept overnight, and consumed orally.	Asthma	2	9	22.22	LC
*Chenopodium album* L. (Amaranthaceae) LAB.514	Che alb	Bathua (Konh)	Herb	Leaves	Decoction of leaves is prepared by boiling.	Abdominal pain	3	10	30	LC
*Coriandrum sativum* L. (Apiaceae) LAB.517	Cor sat	Cilantro (Daniwal)	Herb	Whole plant	Decoction.	Inflammation	2	8	25	-------
*Clinopodium umbrosum* (M.Bieb) Kuntze. (Lamiaceae) LAB.525	Cli umb	Shady calamint (Kunal)	Herb	Aerial part	Aerial parts are boiled in water for half an hour, the obtained water is cooled and used.	Post-delivery bath	3	15	20	NE
*Cynodon dactylon* (L.) Pers (Poaceae) LAB.529	Cyn dac	Bermuda Grass (Dramun)	Herb	Leaves	The fresh leaves are dried, crushed into powder, and consumed in small quantity with water.	Menstrual disorders	5	11	45.45	NE
*Cynoglossum wallichii* G.Don. (Boraginaceae) LAB.530	Cyn wal	Forget Me Not (Cheur)	Herb	Roots	Roots are made into a paste and applied topically.	Skin irritation	3	9	33.33	CR
*Capsella bursa-pastoris* Medik. *LAB.533*	Cap bur	Shepherd’s purse (Kralmond)	Herb	Leaves	Leaves are made into a paste and applied topically.	Inflammation	2	8	25	LC
*Dipsacus inermis* Wall. *(Caprifoliaceae)* LAB.215	Dip ine	Himalayan Teasel (Wopal haakh)	Herb	Leaves	Leaves are crushed and boiled.	Used for bath after delivery	2	7	28.57	NT
*Dysphania botrys* (L.) Mosyakin & Clemants (Amaranthaceae) LAB.519	Dys bot	Sticky goosefoot (Kukli-hakh)	Herb	Whole plant	Decoction.	Labor pain	5	13	38.46	LC
*Dioscorea deltoidea* Wall. (Dioscoreaceae) *LAB.216*	Dio del	Yam (Kala ganda)	Herb	Stem	Stem is crushed and tonic is prepared.	Menstrual cramps	1	7	14.28	EN
*Dryopteris juxtaposita* Christ (Dryopteridaceae) *LAB.217*	Dry jux	Wood Ferns (Gautheer)	Herb	Leaves	Leaves are crushed and boiled.	Used for bath after delivery	2	6	33.32	LC
*Dactylorhiza hatagirea* (D.Don) Soó (Orchidaceae) *LAB.509*	Dac hat	Himalayan Marsch Orchid (salam panj)	Herb	Tuber	Tuber is crushed to powder and given orally with lukewarm water.	Labor pain	5	13	38.46	EN
*Dolomiaea macrocephala* DC. ex Royle (Asteraceae) LAB.222	Dol mac	Dhoop Lakkad (Dupha/Thandi Jaid)	Herb	Root	Roots are boiled, made into a decoction, and taken orally.	Fever	2	8	25	EN
*Euphorbia wallichii* Hook.f. (Euphorbiaceae) LAB.534	Eup wal	Wallich Spurge (Guri-dud)	Herb	Leaves	Infusion is prepared and consumed orally.	Skin irritation	6	14	42.85	LC
*Erigeron canadensis* L. (Asteraceae) LAB.528	Eri can	Horseweed (Shallut)	Herb	Whole plant	Decoction is prepared by boiling the whole plant for 15–20 min, cooled and taken orally.	Menstrual disorders	2	14	14.28	NE
*Eryngium planum* L. (Apiaceae) LAB.543	Ery pla	Sea Holly (Dawha Mool)	Herb	Roots	Dried roots are crushed to increase urine output and thus purify the blood.	Diuretic	2	10	20	LC
*Fragaria nubicola* Lindl. ex Lacaita (Rosaceae) LAB.535	Fra nub	Indian Strawberry (Ringrish)	Herb	Rhizome	Infusion is prepared and consumed orally.	Lactation problem	2	11	18.18	NE
*Ficus carica* L. (Moraceae) LAB.218	Fic car	Fig (Anjeer)	Tree	Fruit	Fruits are dried crushed, made into powder, and taken orally with milk.	Lactation problem	3	8	37.5	LC
*Fumaria indica* (Hausskn.) Pugsley (Fumariaceae) LAB.219	Fum ind	Fumitory (Shahtaur)	Herb	Whole plant	The whole plant is crushed and a decoction is prepared.	Menstrual issue	2	9	22.21	EN
*Fritillaria cirrhosa* D.Don (Liliaceae) LAB.547	Fri cir	Himalayan Fritillary (Sheethkhaar)	Herb	Roots	Fresh roots are crushed and mixed with water and consumed orally.	Abdominal pain	2	13	15.38	EN
*Geranium wallichianum* D.Don (Geraniaceae) LAB.220	Ger wal	Rattan jot (Ratanjot)	Shrub	Root	Roots are made into tea.	Premature delivery	1	5	20	CR
*Glycyrrhiza glabra* L. (Fabaceae) LAB.527	Gly cyr	Black sugar/Sweet wood (Shangir)	Herb	Seeds	Seeds are crushed to form a paste which is applied topically in the lumbar region of the back.	Labor pain	4	15	26.26	LC
*Hemerocallis fulva* (L.) L. (Xanthorrhoeaceae) LAB.221	Hem ful	Common-dayLily (NA)	Herb	Whole plant	The whole plant is crushed and boiled to form a tonic.	Weakness	1	6	16.66	LC
*Isodon rugosus* (Wall.) Codd (Lamiaceae) LAB.521	Iso vul	Wrinkled Leaf Isodon (Sulikath)	Herb	Leaves	Raw leaves are consumed.	Fertility disorder	2	7	28.57	LC
*Juglans regia* L. (Juglandaceae) LAB.223	Jug reg	Walnut (Doen Kul)	Tree	Fruit	Consumed raw.	Fertility issue	7	13	53.84	LC
*Jacobaea analoga* (DC.) Veldkamp (Asteraceae) LAB.556	Jac ana	Ghopga (Boungh)	Herb	Whole plant	Decoction is prepared and consumed orally.	Urinary tract infection (UTI)	2	13	15.38	NE
*Malva cachemiriana* (Cambess.) Alef. (Malvaceae) LAB.551	Mal cac	Kashmir Mallow (Sazposh)	Herb	Seed	Decoction is prepaid and consumed orally.	Skin irritation	2	13	15.38	EN
*Malva sylvestris* L. (Malvaceae) LAB.518	Mal syl	Common mallow (Gur-Sochal)	Herb	Flowers	Decoction.	Weakness	2	8	25	LC
*Meconopsis aculeata* Royle (Papaveraceae) LAB.520	Mec acu	Blue poppy (Gul-e-Neelam)	Herb	Whole plant	Powdered and consumed with water.	Tonic	3	8	37.50	NE
*Mentha arvensis* L. (Lamiaceae) LAB.541	Men arv	Corn Mint (Chala Pudna)	Herb	Leaves	Leaves are dried and made into powder, consumed orally in small quantities.	Vomiting	3	8	37.50	LC
*Mentha longifolia* (L.) L. (Lamiaceae) LAB.542	Men lon	Horse Mint (Jangli Pudina)	Herb	Leaves	Leaves are dried and made into powder, consumed orally in small quantities.	Vomiting	2	6	33.33	LC
*Notholirion thomsonianum* (Royle) Stapf (Liliaceae) LAB.224	Not tho	Rosy Himalayan Lily (Sathra)	Herb	Stem	Stem is crushed and extracted.	Menstrual issue	3	11	27.27	LC
*Nepeta cataria* L. (Lamiaceae) LAB.522	Nep.cat	Catnip (Brair-Ghass)	Herb	Leaves	Leaves are dried and made into powder which is given orally in small quantities.	Vomiting	4	11	36.3	LC
*Origanum vulgare* L. (Lamiaceae) LAB.225	Ori vul	Oregano (Babur)	Herb	Whole plant	The whole plant is boiled in water.	Used for bath after delivery	2	13	15.38	LC
*Oxalis corniculata* L. (Oxalidaceae) LAB.523	Oxa cor	Creeping wood sorrel (Chuk-chin)	Herb	Leaves	Leaves are dried and made into powder which is given orally in small quantity.	Vomiting	3	11	27.27	NE
*Ocimum basilicum* L. (Lamiaceae) LAB.531	Oci bas	Basil (Baber)	Herb	Leaves	Infusion is made from fresh leaves and consumed orally.	Labor pain	3	8	37.50	------
*Pinus roxburghii* Sarg. (Pinaceae) LAB.226	Pin rox	Chir Pine (Chad)	Tree	Seeds	Seeds are roasted.	Weakness	5	12	41.66	LC
*Prunella vulgaris* L. (Lamiaceae) LAB.227	Pru vul	Self-Heal (Kalvuth)	Herb	Whole plant	The whole is boiled in water, and obtained water is used for bathing.	Fever Body pain	6	9	66.65	LC
*Portulaca oleracea* L. (Portulacaceae) LAB.526	Por ole	Common Purslane (Nuner)	Herb	Leaves	Leaves are made into a decoction and consumed orally.	Diuretic	3	11	27.27	NE
*Phytolacca acinosa* Roxb. (Phytolaccaceae) LAB.701	Phy aci	Indian Pokeweed (Haputbrand)	Herb	Fruit	Fruits are crushed, squeezed, and applied topically.	Inflammation	2	7	28.57	NE
*Plantago major* L. (Plantaginaceae) LAB.536	Pla maj	broadleaf plantain Waybread (Bod Gull)	Herb	Leaves	Decoction is made from fresh leaves and consumed orally.	Inflammation	1	7	14.28	LC
*Plantago lanceolata* L. (Plantaginaceae) LAB.537	Pla lan	Narrow Leaf Plantain (Gull)	Herb	Leaves	Leaves are cooked and consumed frequently in the last month of the pregnancy.	Delivery issue	2	9	22.22	VU
*Prunus persica* (L.) Batsch (Rosaceae) LAB.538	Pru per	Peach (Chenan)	Tree	Leaves	Infusion is made and consumed orally.	Fertility disorder	2	8	25	NE
*Podophyllum hexandrum* Royle (Berberidaceae) LAB.539	Pod hex	Himalayan May Apple (Banvagan)	Herb	Whole plant	Infusion is made and consumed orally.	Fertility disorder	1	6	16.66	EN
*Persicaria hydropiper* subsp. *microcarpa* (Danser) Soják (Polygonaceae) LAB.540	Per hyd	Water Pepper (Marchuwagun Ghass)	Herb	Whole plant	Infusion is made and consumed orally.	Labor pain	3	10	30	LC
*Papaver somniferum* L. (Papaveraceae) LAB.552	Pap som	Opium (khash- khaash)	Herb	Seed	The seeds are crushed into powder and consumed in small quantities with milk.	Weakness	3	9	33.33	NE
*Rheum webbianum* Royle (Polygonaceae) LAB.553	Rhe web	Indian Rhubarb (Pambchalan)	Herb	Roots	Crushed roots are mixed with ash and applied topically.	Skin irritation	3	15	20	NE
*Rubus niveus* Thunb. (Rosaceae) LAB.548	Rub niv	Hill Raspberry (Chhanch)	Shrub	Roots	Roots are shade dried and made into powder, consumed with milk in the last month of pregnancy.	Delivery problem	2	15	13.33	NE
*Sonchus oleraceus* L. (Asteraceae) LAB.228	Son ole	Sow Thistle (Kulwauth)	Herb	Leaves	Leaves are cooked.	Weakness	1	5	20	LC
*Saussurea simpsoniana* (Fielding & Gardner) Lipsch. (Asteraceae) LAB.230	Sau sim	Phen Kamal (Koth)	Herb	Whole plant	Whole plant is crushed and boiled in water taken orally.	Menstrual issue	3	7	42.85	CR
*Salvia sclarea* L. (Lamiaceae) LAB.507	Sal scl	Clary sage (Buder-Tund)	Herb	Whole plant	Decoction is obtained and given orally.	Labor Pain	3	8	37.5	LC
*Sisymbrium irio* L. (Brassicaceae) LAB.555	Sis iri	London Rocket (Chari lachij)	Herb	Seeds	Decoction is prepared and consumed orally.	Asthma	4	11	36.36	LC
*Taraxacum officinale* F.H.Wigg. (Asteraceae) LAB.231	Tar off	Dandelion (Handd)	Herb	Leaves	Young leaves are cooked.	Easy delivery	5	7	71.42	LC
*Trifolium pratense* L. (Fabaceae) LAB.232	Tri pre	Red Clover (Bee-Bred)	Herb	Leaves	Leaves are crushed, dried, made into powder, and consumed with milk.	Menstrual issue	1	9	11.10	LC
*Trigonella foenum-graecum* L. (Fabaceae) LAB.508	Tri foe	Fenugreek (Meth)	Herb	Seeds	Seeds are crushed into powder and consumed with water.	Menopause disorders	2	8	25	-----
*Thymus linearis* Benth. (Lamiaceae) LAB.544	Thy lin	Himalayan Thyme (Van Jawain, Marchi)	Herb	Whole plant	Juice is extracted from the whole plant and consumed orally.	Urinary tract infection (UTI)	3	12	25	CR
*Tagetes minuta* L. (Asteraceae) LAB.554	Tag min	Wild Marigold (Gutt posh)	Herb	Leaves	Juice is extracted and consumed orally.	Asthma	2	15	13.33	NE
*Viola biflora* L. (Violaceae) LAB.545	Vio bif	Alpine Yellow Violet (Gulnakash)	Herb	Leaves	Decoction is prepared and consumed orally.	Fever	2	14	14.28	NE
*Viola odorata* L. (Violaceae) LAB.546	Vio odo	Sweet Voilet (Nunposh/Banfsha)	Herb	Flowers	Decoction is prepared and consumed orally.	Fever	4	15	26.26	LC
*Verbena officinalis* L. (Verbanaceae) LAB.524	Ver off	Common Verbena (Hatmool)	Herb	Whole plant	Whole plant is crushed to powder and given orally with water in the last month of pregnancy.	Delivery issue	2	9	22.22	NE
*Viburnum grandiflorum* Wall. ex DC. (Adoxaceae) LAB.549	Vib gra	Cranberry Bush (Kilmish)	Shrub	Roots	Roots are boiled in water and consumed orally.	Abdominal pain	2	11	18.18	NE

LC: Least Concern; CR Critically Endangered; EN: Endangered; NT: Near Threatened; VU: Vulnerable; TH: Threatened; DD: Data Deficient. Np: Number of informants reporting species used for specific disease; FL: Fidelity level; FC: Frequency of Citation; Abre: Abbreviation.

### 2.3. Fidelity Level (FL)

In the present study, the species most preferred for the treatment of specific ailments were identified by calculating FL. According to Farooq et al. [29], the species that are most commonly used medicinally in certain areas have a maximum FL. In calculating FL for our results, it was found to be 11.10 to 71.42 (Table 2). The highest value was calculated for *Taraxacum officinale* (71.42) for easy delivery, followed by *Allium sativum* (62.5) for conception problems, *Juglans regia* (53.84) for fertility problems, and *Cydonia oblonga* (44.43) for constipation. The lowest value was calculated for *Trifolium pretense* (11.10) for irregular menstruation. In the Kashmir Himalayas (in most ethnic groups such as Kashmiri, Gujjar, Pahari), *Taraxacum officinale* is considered an important traditional medicine given to woman for easy childbirth and other gynecological problems [15]. The phytochemistry of *Taraxacum officinale* also confirms the presence of various essential chemical constituents such as sesquiterpenes, lactones, fatty acids, carotenoids, tannins, carbohydrates, phenolic acids, flavonoids, phytosterols, sugars, triterpenes, calcium, proteins, and minerals which are important for the new woman or new mothers [30].

### 2.4. Part Used

**Figure 4:**
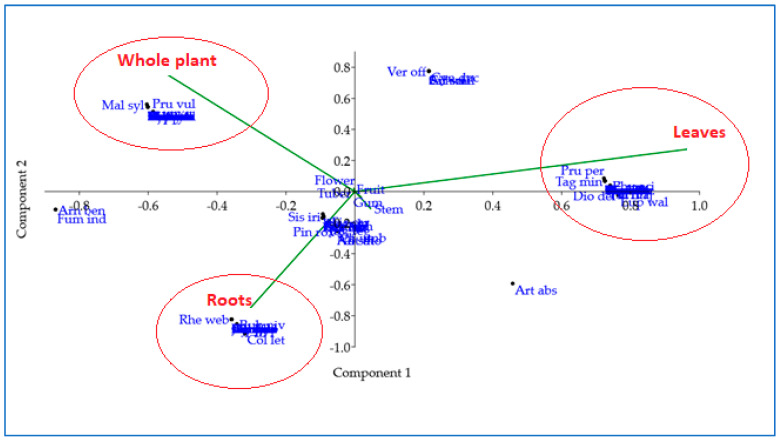
Principal component analysis (PCA) biplot of the different parts of the plant used.

The use of the different parts of the plant for ethnomedicinal purposes showed statistically significant differences (χ^2^ = 90.587, df = 7, *p* < 0.001) in the respective applications. Leaves were the most commonly used, accounting for 30% of the total use. Notable species associated with this preference were *Artemisia absinthium*, *Centaurea iberica*, *Cichorium intybus*, *Dipsacus inermis*, *Dryopteris juxtaposita*, *Sonchus oleraceus*, *Taraxacum officinale*, and *Trifolium pretense*. After that, the whole plant accounted for 25% of use with species such as *Arnebia benthamii*, *Asplenium falcatum*, *Adiantum venustum*, *Fumaria indica*, *Hemerocallis fulva*, *Origanum vulgare*, *Prunella vulgaris*, and *Saussurea simpsoniana*. The roots of the “whole plant” followed with a usage rate of 17%, with species such as *Arnebia benthamii*, *Colchicum leteum*, *Fumaria indica*, *Geranium wallichianum*, *Jurimea dolomiaea*, *Aucklandia costus*, *Aconitum laeve*, *Achyranthes aspera*, *Astragalus confertus*, *Aquilegia fragrans*, *Cynoglossum wallichii*, and *Ocimum basilicum* being important in this context. Principal component analysis (PCA) [Figure 4] also confirmed the distinction between the “whole plant”, leaves, and roots, while the remaining plant parts were grouped together. The predominant use of leaves can be attributed to their ease of collection and the belief in the presence of various phytocomponents [31]. In addition, leaves are commonly used in traditional medicine to alleviate a variety of ailments [32].

### 2.5. Gastronomic Usage

**Figure 5:**
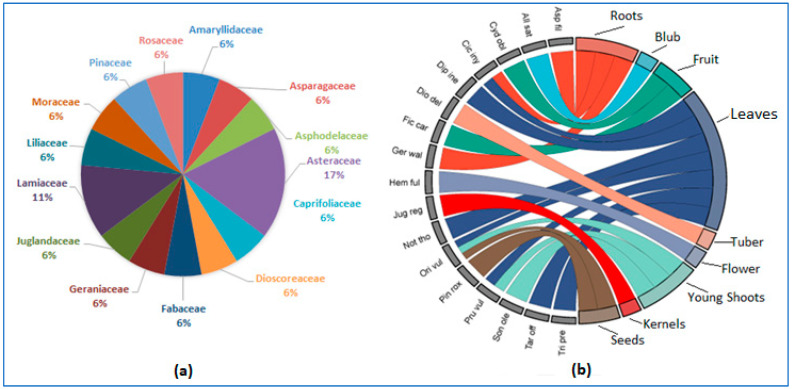
(**a**) Percentage of families with gastronomic assignment. (**b**) Chord diagram showing the different parts of the documented species for gastronomic use.

Indigenous communities living in the Himalayan region have traditional cultural practices for using local edible plant species [33]. From the documented species, only *n* = 17 (herbs *n* = 13; trees *n* = 4) were recorded for gastronomic use, belonging to *n* = 14 families, representing 56.6% of the total documented species. Asteraceae (17%) was the dominant family, followed by Lamiaceae (11%) (Figure 5a). The above species are further divided into wild and cultivated species. The wild species (*n* = 13) outweighed the cultivated ones (*n* = 4) (Table 2). This clearly shows the importance of wild species in the region. Haq et al. [15] studied the importance of wild foods in the Kashmir-Himalayan region shared by India, Pakistan, and China and found that wild foods are a boon in the rural areas of the region and people there are very attached to them due to their proximity to nature and lack of urbanization. Leaves (*n* = 7) were the most commonly consumed, followed by young shoots, roots (*n* = 3 each), seeds, and fruits (*n* = 2 each). Azhar et al. [34] reported the predominance of the leaves in traditional medicine from southern Punjab, Pakistan. Similar results were reported by Mir et al. [35] from the Himalayan region of Kashmir. Figure 5b shows the parts with gastronomic uses and the corresponding species. A complete list can be found in Table 2.

**Table 2:** Plant species consumed by the Dard community in the Kashmir Valley with respect to pregnancy.

Botanical Name (Abbreviation)	Species Consumed			Preparation	Habitat
	Before Pregnancy	During Pregnancy	After Pregnancy		
*Asparagus filicinus* (Asp fil)	Y	Y	Y	Young roots are selected and made into soup.	Wild
*Allium sativum* (All sat)	Y	Y	N	Bulbs are peeled, cleaned, and cooked with other vegetables. Raw bulbs are consumed as a salad.	Cultivated
*Cydonia oblonga* (Cyd obl)	Y	Y	N	The fruits are boiled in water with a little sugar added and eaten in the evening.	Cultivated
*Cichorium intybus* (Cic int)	N	Y	Y	The leaves are picked, cleaned, and cooked without species and peppers.	Wild
*Dipsacus inermis* (Dip.ine)	N	N	Y	The leaves are picked, cleaned, and cooked without species and peppers.	Wild
*Dioscorea deltoidea* (Dio del)	Y	N	N	Tubers are boiled and then cooked without species.	Wild
*Ficus carica* (Fic car)	Y	Y	Y	Fruits are consumed raw, also cooked.	Cultivated
*Geranium wallichianum* (Ger wal)	N	Y	N	The roots are cooked in desi cow ghee.	Wild
*Hemerocallis fulva* (Hem ful)	Y	Y	Y	The flowers are eaten raw, but also cooked. The petals are thick and crunchy, which makes them very pleasant to eat.	Wild
*Juglans regia* (Jug reg)	Y	Y	Y	The seeds are extracted and used to make chutney, but they are also air-dried and eaten raw. In addition, they are also used in kawa (a traditional tea) mixed with various recipes to enhance the flavor. It is also a possible source of cooking oil.	Cultivated
*Notholirion thomsonianum* (Not tho)	Y	N	N	Leaves are boiled and then cooked.	Wild
*Origanum vulgare* (Ori vul)	N	N	Y	Fresh oregano leaves are cooked. Dried leaves are used as a flavoring agent for different dishes.	Wild
*Pinus roxburghii* (Pin rox)	N	N	Y	Seeds are roasted and eaten.	Wild
*Prunella vulgaris* (Pru vul)	N	N	Y	The leaves are used for making soup, stews, salad and boiled as a pot herb.	Wild
*Sonchus oleraceus* (Son ole)	N	Y	Y	The outer shell is removed and cooked like asparagus.	Wild
*Taraxacum officinale* (Tar off)	Y	Y	Y	The leaves are cleaned and cooked.	Wild
*Trifolium pratense* (Tri pra)	Y	Y	Y	`The leaves are cooked or used as a garnishing agent in salads.	Wild

### 2.6. Use Value (UV)

**Figure 6:**
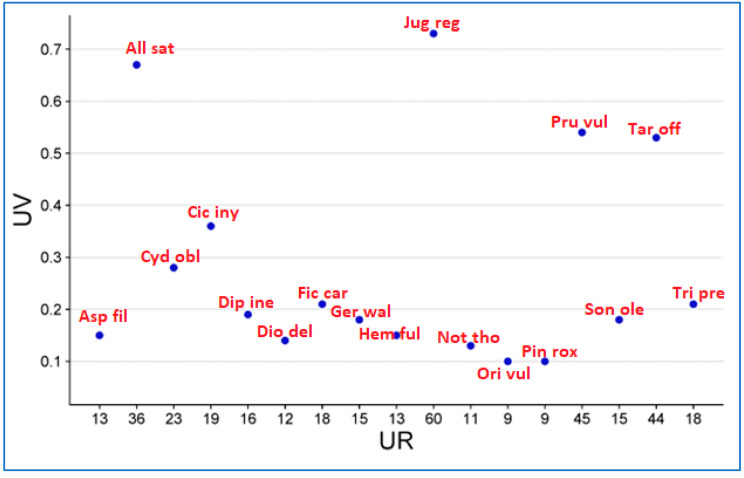
Association between use value (UV) and use reports for the species with gastronomic assignment. The full name of the species is given in Table 3.

When analyzing the results documented for species consumed as food before pregnancy (after marriage), during pregnancy, and after pregnancy, the UV ranged from 0.10 to 0.73 (Figure 6). The highest value was calculated for *Juglans regia* (0.73), followed by *Allium sativum* (0.67), *Prunella vulgaris* (0.54) and *Taraxacum officinale* (0.53). Species with the highest use values (UVs) had the highest level of awareness, while species with lower UVs had a correspondingly lower level of awareness. *Juglans regia* has many uses in cooking. It is often mixed with other species such as mint to make chutney. It also plays an important role in the preparation of a traditional hot beverage known as “kawa”. Many people extract oil from it, which in turn is used in cooking for special recipes such as *yaja* (made from rice flour), as shown in Figure 2b. In addition, the seeds are often incorporated into various dishes such as *biryani* and *salt tea* to give them a better flavor profile.

They are also a possible source of cooking oil. *Allium sativum* is also consumed as a spice, especially in combination with a variety of vegetables to enhance flavor. It is believed to nourish the gastrointestinal tract, and due to its aromatic nature, locals prefer to consume garlic because they believe it has the potential to warm the body in the cold, long winter. According to [36], *Allium sativum* has been successfully used as food and medicine in human societies since ancient times. *Prunella vulgaris* is mainly consumed as soup and is said to restore strength after childbirth, with some olive oil added to the soup to lubricate the digestive tract for good absorption. *Taraxacum officinale* is also considered a nutrient-rich food, as it contains a variety of components that are vital for pregnant women. The presence of chemical constituents such as carbohydrates, calcium, proteins, and minerals makes the species a potent gastronomic candidate [30].

In the present study, we analyzed the use of plant species based on the preference (during, after, and before pregnancy) and seasonal availability for gastronomic use. The results (Table 2) show that *n* = 3 species *(Pinus roxburghii*, *Dipsacus inermis*, *Origanum vulgare*) were the only ones preferred “*after pregnancy usage*” followed by *n* = 2 species (*Notholirion thomsonianum*, *Dioscorea deltoidea*) “*before pregnancy”*, and only *n* = 1 (*Geranium wallichianum*) “*during pregnancy”*. When the frequently consumed (during, after, and before pregnancy) were examined, a total of *n* = 6 species *(Ficus carica*, *Trifolium pratense*, *Hemerocallis fulva*, *Asparagus filicinus*, *Juglans regia*, and *Taraxacum officinale)* were detected.

### 2.7. Novelty of the Study

By comparing our research results with previous studies conducted in the nearby region [8,18,19,26,31,33], we discovered new applications in the field of ethnomedicine. For example, we found that the seeds of *Asparagus filicinus* are used to relieve physical ailments caused by postpartum weakness. Similarly, the roots and stems of *Geranium wallichianum* are used to treat preterm labor and breastfeeding. In addition, *Alcea rosea* shows effectiveness in treating skin irritation, while *Arnebia benthami* (used in its entirety) is known for its usefulness for breastfeeding problems of new mothers. It is worth noting that certain pious people, referred to as Pir and Baba, get enchanted with *Arnebia benthamii* before using it to treat breastfeeding problems. It is also important to note that only one previous study [26] reported the use of plant species in the Dard community. The Jaccard similarity index is 11.49, but no similarity was found between the species used in the present study. In addition, the sample size of the previous study is very small (*n* = 35) compared to that of the present study (*n* = 82), and the study area is different.

### 2.8. Effective Use and Livelihood

In the Kashmir Valley, agriculture and related sectors are the main source of livelihood for the majority of the population [7]. Our research efforts have led to the identification of a variety of plant species, including *Prunus persica*, *Allium sativum*, *Ficus carica*, *Juglans regia*, *Mentha arvensis*, and *Ocimum basilicum*, all of which have a significant impact on the local economy due to their substantial contribution to food habits.

*Juglans regia*, for example, is known for its high economic value and has led to a thriving cottage industry in which numerous individuals extract the tree’s seeds and export them to various regions of the country, resulting in significant economic gains. *Allium sativum* is considered an indispensable spice in the region’s culinary repertoire and remains readily available in local markets. *Prunus persica* is mainly dried and exported to other parts of the country. *Ocimum basilicum* is highly valued in the region, mainly because it facilitates fast-breaking, and its availability in the market contributes significantly to the local economy. In addition, the systematic processing of *Mentha arvensis* plays a central role in the production of various local spices and dips, which brings significant economic benefits to the region.

### 2.9. Conservation Status

In the IUCN conservation assessment of the documented species, *n* = 7 species (*Aquilegia fragrans*, *Arnebia benthamii*, *Cynoglossum wallichii*, *Geranium wallichianum*, *Aucklandia costus*, *Saussurea simpsoniana*, *and Thymus linearis*) are classified as critically endangered (CR), *n* = 10 species (*Adiantum venustum*, *Artemisia absinthium*, *Aconitum leave*, *Dioscorea deltoidei*, *Dactylorhiza hatagirea*, *Fumaria indica*, *Fritillaria cirrhosa*, *Jurinea dolomiaea*, *Malva cachemiriana*, *and Podophyllum hexandrum*) as endangered (EN), and *n* = 4 species *(Asplenium falcatum*, *Ajuga parviflora*, *Cannabis sativa*, and *Plantago lanceolata*) as part of the vulnerable category (VU) (Table 1). Many species with medicinal importance are threatened on a large scale due to the extensive use of their required parts. Because of their medicinal importance, these species have developed an economic value for which many people over- collect, which has promoted the decline of species throughout the Himalayan region [37]. At the same time, the global phenomenon of urbanization is driving development initiatives that include road expansion and building construction, increasing the influx of people and consequently creating the potential for new threats.

Gopi et al. [38] reported that nearly 15,000 plant species used in traditional medicine worldwide fall into the category of endangered species and therefore require immediate conservation and mitigation measures to ensure their survival. Many ethnic groups have potential ecological knowledge that, if used scientifically, can contribute to the sustainable use of biological species, which in turn will contribute to the conservation of eroding green wealth. Currently, there is more and more discussion around the world about community-based conservation that incorporates not only includes the species, but also the concerns (local values, local beliefs) of local people.

## 3. Materials and Methods

### 3.1. Study Area

**Figure 7:**
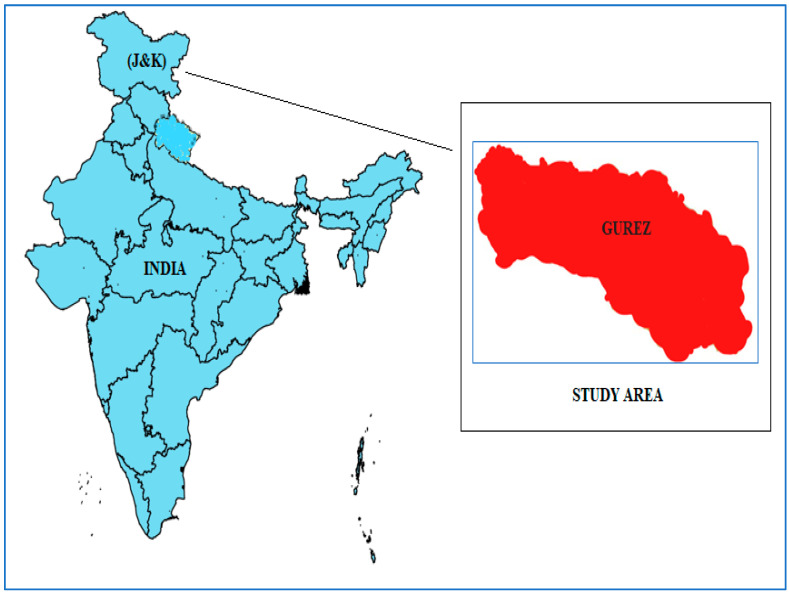
Map of the study area (Gurez), Jammu and Kashmir (J&K), India.

Gurez, also known as Gurais (Figure 7), is located in the Himalayan region, about 123 km north of Srinagar (Jammu and Kashmir, India). The valley is located near the Pakistan Line of Control and is at an elevation of about 2438 m above sea level. The area is cut off from the outside world for almost six months of the year due to heavy winter snowfall (2 m). Nearly 30,000 people live in the area, distributed among fifteen communities. The Kishanganga River flows through the area and provides irrigation. The different physical characteristics provide many habitats and microhabitats where a variety of plant species thrive, while oak, Betula, Cedrus, and Pinus are also important species in the forests, whose vegetation begins in late summer [19]. The Gurez Valley is characterized by a rugged mountainous landscape. Climatic conditions in the valley follow a temperate pattern influenced by a variety of topographic features that result in relatively mild summers and severe winters. The warmest and coldest seasons in this region are July and December, respectively. In addition, the valley records its highest rainfall during the months of March and April [7].

According to the last census, most of the people are Muslims (83.98%), followed by Hinduism (14.24%), Sikhism (1.16%), Christianity (0.36%), and Buddhism (0.05%) [39]. Ethnic tribes currently living there include Gujjar, Bakarwal, and Kashmiri. Also living in the valley houses are the Dards, who are cut off from their mainland Astore, Gilgit, and Chilas by the Line of Control.

### 3.2. Socio-Economic Background of Dard

*Dards* are the earliest coterie, believed to be the most ancient people of the Aryans who arrived in the Indian subcontinent nearly two thousand years ago. *Kalhana*, a Kashmiri historian (1145 AD) who wrote the “*Rajatarangini*”, also describes them as the descendants of the Aryan race [40]. They spread across the Himalayas, usually choosing to settle in the Hindu Kush mountains until they spread to the lower areas of the Himalayas. Herodotus, a famous Greek poet, mentioned the Dard-Shins in 430 BC, confirming the presence of the Dards in the Himalayan region. In Jammu and Kashmir (J&K), the majority of this community currently lives in the Gurez sub-district of Bandipora (administrative district of Jammu and Kashmir) [41]. The spoken language is Shina, and other languages include Urdu and Kashmiri. The area (Gurez) is economically backward and has few modern facilities. The people (Dard) depend largely on natural resources such as forests for food and firewood; they also engage in livestock rearing, cottage industries, and trade. In the social ranking, the community is divided into four categories (Renue: ruling class, Shins: religious group, Yashkun: farmers/peasants, Dum: lower group).

### 3.3. Demography of Informants

A total of 82 people were selected for the interview, 63 of whom were men and 19 of whom were women. The predominance of men over women was due to cultural constraints. Prior to recording, frequent visits were made to the study to ensure the participation of local people. Documented species of medicinal and gastronomic importance were collected from March to October 2022; a complete summary of the collection is provided in Table 3. Documentation followed the method (snowball technique) used by Haq et al. [23]. Prior to each interview, verbal consent was obtained and the code of ethics was followed (International Society of Ethnobiology, Code of Ethics, 2006) [42]. Interviews were conducted in the native language with the assistance of a translator. The ethnicity of the informant and language information were not disclosed, as required by the Nagoya Protocol. We conducted the interviews with informants of all ages, genders, and occupations. Semi-structured questions were used to capture traditional knowledge [24]. The most knowledgeable people were elderly, and the bulk of respondents (45.12%) were illiterate (Table 3). At least one qualified informant helped to verify the samples and helped with preparing the herbarium at each research site. Flora POWO 2023 was used to authenticate plant names [43].

**Table 3:** Demographic status of the respondents from study area.

Demographic Features	Number	Percentage
**Ethnic Group**	**Dard**	
**Language**	(Shina)	
**Education**		
Illiterate	37	45.12
Primary education	19	23.17
Secondary education	17	20.73
Higher education	9	10.97
**Age range**		
Young (18–26)	15	18.29
Middle (27–55)	26	31.70
Old (56–75+)	41	50.00
**Profession**		
Farmers	12	14.63
Skilled/semi-skilled workers	15	18.29
Grower/agricultural workers	12	14.63
Herders	18	21.95
Government employees	5	6.09
Housewives	8	9.75
Shopkeepers	12	14.63
**Gender**		
Male	63	76.82
Female	19	23.17
**Religion**	Islam	100

### 3.4. Data Analysis

We used a matrix plot employing Paste 4.03 to show the distribution of species among the families. A balloon plot was utilized to represent the species used against different diseases. A cladogram showing the Pearson correlation between plant species and diseases was used (https://www.bioinformatics.com.cn/en?p=5, accessed on 2 October 2022). A chord diagram with a pie chart was used to evaluate the number of plant parts with gastronomic uses by using the circle package in R studio. The same tool was used to show the relationship between UR and UV [24]. Paste software (4.03) was used for principal component analysis (PCA) to show the frequently used plant parts of the documented species against a variety of diseases.

#### 3.4.1. Fidelity Level (FL)

FL was used to calculate the percentage of respondents who reported similar use of the species [24]. We calculated it using the following formula.

(1) $$FL\;\left(\%\right)=\left(\frac{Np}{Fc}\right)\times 100$$

Np is the number of informants reporting the use of a species to treat a particular disease, and Fc (frequency of mentions) is the number of informants reporting the use of a species to treat a particular disease.

#### 3.4.2. Use Value (UV)

To evaluate the proportional value of species use, we used use value indices using the following formula [24].

(2) $$Uv=\frac{\Sigma Ur}{N}$$

“Ur” represents the number of use reports of the use of a particular species, and “N” represents the total number of respondents.

## 4. Conclusions

The current study attempted to acquire information about a little-studied ethnic community (Dard) for the use of plant taxa to treat health disorders and to identify species consumed (before, during, and after pregnancy) as primary food. Living in harsh climatic conditions, the said ethnic community has gained the knowledge of how to use the flora for their primary needs (medicine and food). Unfortunately, traditional knowledge (TK) is primarily limited to the elderly due to the lack of interest among the younger generation. This lack of interest is due to urbanization forcing the people to change their mode of living, i.e., young people migrate to Srinagar (capital city) for education, many for labor, and many have invested in small-scale shops. Hence, the inheritance of TK has been met with a potential pause in the ethnic group. In this regard, it is imperative to document the eroding traditional knowledge before it is completely lost. Many plant species used are on the IUCN’s list of critically endangered, endangered, and vulnerable species; in this regard, local people require proper education to aid in conservation and long-term sustainability. Furthermore, species new to the ethnomedicinal literature should be considered for bioprospecting for the possible discovery of some potent novel molecules with strong medical implications. The current study gives a baseline for understanding the importance of native plant species in the daily lives of the Dard community, which will benefit the local government in developing strategies to help the community’s development. In addition, this study gathers basic information from the Kashmir-Himalayan region that hopefully can be used by pharmaceutical companies to discover new compounds with remarkable therapeutic properties.

## Data Availability

All collected data are provided in the article.
